# Utility and Safety of 5-ALA Guided Surgery in Pediatric Brain Tumors: A Systematic Review

**DOI:** 10.3390/cancers16213677

**Published:** 2024-10-30

**Authors:** Cheng Wang, Ying Yu, Yafei Wang, Jiahua Yu, Chenran Zhang

**Affiliations:** 1 Department of Pediatric Neurosurgery, Xinhua Hospital, Shanghai Jiao Tong University School of Medicine, Shanghai 200092, China; c.wang_trs@sjtu.edu.cn (C.W.); yujiahua.shjd@sjtu.edu.cn (J.Y.); 2 School of Medicine, Shanghai Jiao Tong University, Shanghai 200025, China

**Keywords:** 5-aminolevulinic acid, fluorescence guided surgery, pediatric brain tumor

## Abstract

**Background:** 5-Aminolevulinic acid-guided surgery for adult gliomas has been approved by the European Medicines Agency and the US Food and Drug Administration, becoming a reliable tool for improving gross total resection rates and patient outcomes. This has led several medical centers to explore the off-label use of 5-ALA in the resection of pediatric brain tumors, assessing its efficacy and safety across various tumor types. However, given the differences between children and adults, the appropriateness of 5-ALA use in pediatric populations has not yet been fully established. **Methods:** We collected eligible publications from Embase, Scopus, PubMed, and Proquest, ultimately selecting 27 studies. Data extraction and retrospective analysis of 249 surgical cases were conducted to determine the current efficacy and safety of 5-ALA in pediatric brain tumors. The fluorescence rate and utility stratified by several clinical features, including WHO grade, tumor classification, and tumor location, were analyzed. **Results:** Most studies suggest that 5-ALA can enhance tumor identification in high-grade tumors, including glioblastomas and anaplastic astrocytomas. Changes in survival or recurrence rates associated with 5-ALA-guided resection have not been reported. None of the cases reported significant postoperative complications related to the use of 5-ALA. **Conclusions:** 5-ALA can aid in the resection of high-grade gliomas in pediatric patients. The efficacy of 5-ALA in low-grade gliomas and other tumors may require enhancement with additional tools or modified administration protocols. The safety of 5-ALA has reached a preliminary consensus, although further randomized controlled trials and data on survival and molecular characteristics are needed.

## Simple Summary

In brain tumor surgery, increasing the extent of resection has been shown to improve patient outcomes and survival. 5-ALA serves as a tumor visualization adjunct and has been approved for use in adult high-grade gliomas. The purpose of this systematic review was to assess whether 5-ALA has similar utility and safety in pediatric patients. A total of 249 pediatric cases were identified from the relevant literature, confirming the safety of 5-ALA in this population. Overall, the fluorescence rates and utility were favorable, although there were some variations across different tumor grades and types. While our preliminary findings suggest that 5-ALA is both safe and effective in pediatric brain tumor surgery, further systematic clinical studies are needed to validate these results.

## 1. Introduction

Brain tumors account for 25–30% of all pediatric cancers, making them the second most common type of malignancy in children. Surgical resection is a standard component of treatment, often combined with radiotherapy and chemotherapy, particularly in younger children who are less able to tolerate the side effects of radiation and chemotherapy [1]. The effectiveness of surgical resection, therefore, is a key factor in improving survival rates while minimizing adverse effects. As recommended by the National Comprehensive Cancer Network (NCCN) and other studies, the maximal extent of resection (EOR) of a brain tumor should be performed to achieve prolonged progression-free survival (PFS), prolonged overall survival (OS), and a greater percentage of gross-total resection (GTR) [1,2,3,4,5,6,7,8,9,10,11,12,13,14,15].

In recent years, several tools have been developed to assist in the resection of adult brain tumors, including fluorescent dyes, intraoperative ultrasound, and intraoperative MRI [4,16,17]. Among these, 5-aminolevulinic acid (5-ALA) has received approval from both the European Medicines Agency and the US Food and Drug Administration for the resection in adult patients with suspected high-grade gliomas. 5-ALA serves as an adjunct for visualizing malignant tissue during surgery by accumulating in tumor cells and fluorescing after irradiation with a blue light source (wavelength of 375–410 nm) [4,6,18]. Compared with standard white-light surgery, it has been shown to improve the identification of residual tumor tissue, leading to a greater EOR and a higher rate of gross total resection (GTR). In a phase III clinical trial in adult patients, 5-ALA-guided surgery was associated with an increased gross total resection rate of 65% and a reduced risk of death or progression compared with white light surgery (hazard ratio 0.73 [0.57–0.94], *p* = 0.01) [3]. In a Cochrane meta-analysis on intraoperative imaging technologies for adult glioma resection, 5-ALA, alongside intraoperative MRI, was found to potentially enhance the extent of resection in patients with high-grade glioma [4].

The significant utility of 5-ALA in adults has encouraged its application in pediatric patients. While the safety and efficacy of 5-ALA in pediatric patients have not yet been established through large-scale clinical trials, initial case reports and case series have demonstrated its potential benefits in both high-grade and certain low-grade brain tumors in children. Zhang et al. and Schwake et al. independently reviewed 5-ALA fluorescence-guided surgery in pediatric brain tumors and reported that 5-ALA can facilitate tumor resection, particularly given its favorable safety profile [8,15]. However, despite these advancements, several key technical gaps remain, including limited accuracy in identifying tumor boundaries, inconsistencies in resection outcomes, and the lack of robust tools for assessing the severity of tumor progression. Moreover, due to the limited sample sizes, further data analysis has not been conducted. Over the past five years, the number of pediatric cases has steadily increased, although still limited to a handful of cases. A large multicenter study has not been performed to date.

In this study, we conducted a systematic review of the existing literature on 5-ALA-guided surgery in pediatric brain tumors. We performed a comprehensive analysis of the available data, focusing on resection rates, neurological outcomes, and intraoperative findings. Our systematic review follows the PRISMA guidelines, and relevant metrics were collected and analyzed to provide up-to-date recommendations. This study aims to address the current gaps in the literature and offer insights into performance differences and influencing factors in pediatric 5-ALA-guided surgery.

## 2. Materials and Methods

### 2.1. Search Strategy

A search based on the Preferred Reporting Items for Systematic Reviews and Meta-Analyses (PRISMA) statement was conducted in databases including Embase, PubMed, Scopus, and Proquest to identify publications on fluorescence-guided surgery with 5-ALA in pediatric brain tumors [19]. All publications up to 1 August 2024 were included without any restriction of language or article type. For the search, we used medical subject terms and keywords to search for titles and abstracts. The review was not registered.

**Figure 1:**
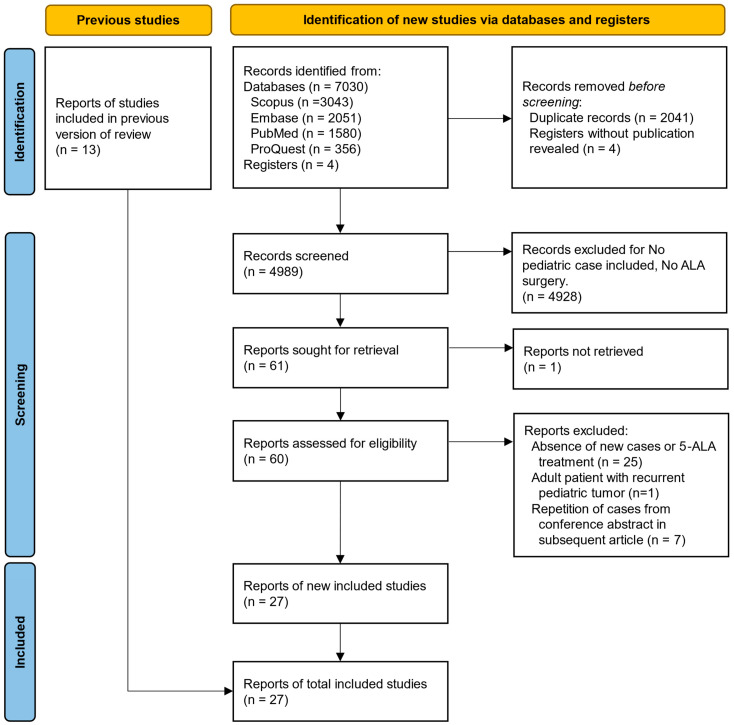
Flow diagram for updated systematic reviews, including searches of databases, registers, and other sources. (PRISMA 2020).

The search strategy was based on the PICO model (the initials “patient”, “intervention”, “control”, “outcome”) and consisted of children AND 5-aminolevulinic acid AND brain tumor. To maximize the systematicity of all pediatric patient reports within articles that were primarily focused on adult cases, we did not choose to limit the initial search to “pediatric* OR child*” terms. Studies were included if they met the following criteria: (1) studies on surgeries of brain tumors or brain metastases; (2) studies that have utilized 5-ALA as a fluorescence adjunct. We subsequently excluded studies without any pediatric case (under 18 years old). The search strategies and the search terms can be accessed in Supplementary Table S1. This study selection process is presented in Figure 1 using the PRISMA flow diagram.

One author (C.W.) initially screened the titles and abstracts of the articles retrieved from the previous search to determine if they met the inclusion criteria with Microsoft Excel 2021 (Version 16.88). If a study was considered potentially eligible, the full text was then reviewed to confirm or exclude. The search process was supervised by another author (C.Z.). Next, relevant articles were retrieved and evaluated independently by two authors (C.Z. and Y.W.) using Zotero (Version 6.0.37, Corporation for Digital Scholarship, Vienna, VA, USA). Some irrelevant studies included in previous reviews were excluded because they did not involve pediatric cases [20]. To avoid unreliable statistics due to duplicated data, cases with similar collection periods and characteristics were treated as duplicates, particularly those reported in conference abstracts.

A cross-reference check of the citations of each relevant literature review included was performed to ensure that no relevant studies were missed by the computed database search. Four clinical trial registrations were found as 5-ALA with pediatric brain tumors on clinicaltrial.gov (NCT05123534, NCT04738162, NCT02050243, and NCT00671710). No associated articles were found from the clinical trial.

### 2.2. Data Extraction and Risk of Bias Assessment

Two authors (C.W. and Y.W.) extracted data separately in Microsoft Excel sheets, which were then cross-checked to ensure accuracy. The data collected included population size, age in years, number of males, tumor classification, WHO grade, 5-ALA dose (mg/kg), tumor location (supratentorial or infratentorial), examination, extent of resection, complications, usefulness, and follow-up duration. We followed the 2007 WHO brain tumor classification since the molecular types were not available in most reports. Some cases were repeatedly reported in subsequent retrospective studies and were identified and excluded based on the timing, source of reporting, and case characteristics. Those duplicate cases were manually merged with complementary descriptions found in different reports. Two authors (C.W. and Y.Y.) accessed the risk of bias and quality of studies independently with the Joanna Briggs Institute (JBI) Critical Appraisal Checklists for Case Reports and Case Series [21,22].

### 2.3. Data Analysis

Descriptive statistics were used to describe the data by Microsoft Excel and GraphPad Prism (Version 10.2.0, GraphPad Software, Boston, MA, USA). Statistical analyses and meta-analyses could not be performed because of the limited sample size of the articles and patients and the heterogeneity of the data.

## 3. Results

### 3.1. Summary of Search Results

Twenty-seven studies were included after applying the inclusion and exclusion criteria (Figure 1). The included studies were published between 2009 and 2024 (until 1 August 2024). The studies included 249 patients (307 reports, patients from Preuß et al., Kim et al., Agawa et al., and Sysoev et al. were repeated in subsequent studies) who underwent 5-ALA-guided brain tumor resection, of which 55 of 115 (47.8%) were females. The overall age of this study population was 8.59 ± 4.64 years (the age of 74 patients was unknown). The detailed demographic characteristics of the included studies are shown in Table 1. The quality assessment details are shown in Supplementary Table S2.

Reports were almost equal in supratentorial (107, 52.2%) and infratentorial (98, 47.8%) from 205 cases reported. Pilocytic astrocytomas (PCAs) were the most commonly reported type, accounting for 16.9% of cases, followed by medulloblastomas at 16.0%, glioblastomas (GBMs) at 13.3%, and ependymomas (Eps) at 10.0%. A summary of the fluorescence grade, EOR, utility of fluorescence, and location in different tumors is presented in Table 2.

### 3.2. Fluorescence Rate and Utility

**Figure 2:**
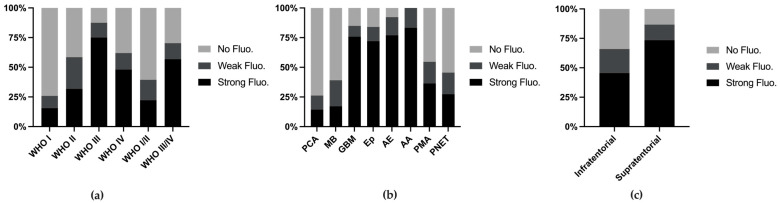
Fluorescence rate stratified by WHO grade (**a**), tumor classification (**b**), and tumor location (**c**).

We combined various metrics from the reports and conducted analyses. Among all 249 cases (with 2 cases missing fluorescence data), the fluorescence rates increased with higher WHO grades (Figure 2a). When stratifying tumor types with more than 10 reported cases, we found that the fluorescence rates exceeded 50% in anaplastic astrocytomas (83.2%), anaplastic ependymomas (76.9%), ependymomas (76.0%), and glioblastomas (75.8%), whereas common tumors such as PCA (14.3%) and MB (17.1%) presented lower fluorescence rates (Figure 2b). Supratentorial tumors presented higher fluorescence rates (73.3%), which can be attributed to the greater incidence of MB and PA, both of which present lower fluorescence rates, in the posterior fossa (Figure 2c).

**Figure 3:**
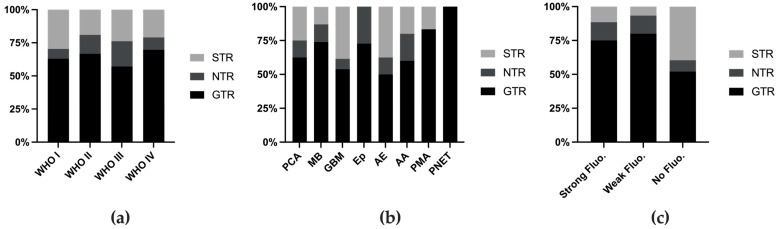
Extent of resection rate stratified by WHO grade (**a**), tumor classification (**b**), and fluorescence intensity (**c**). Fluo. fluorescence.

The definition of usefulness varies across different reports. In 2014, Stummer defined usefulness as a change in surgical strategy or tumor identification on the basis of 5-ALA fluorescence [26], whereas subsequent studies did not specify whether their definition met this standard or was based on subjective assessment. Therefore, we assessed the extent of resection (EOR) as a measure of utility. The EOR was generally similar across different tumor grades (Figure 3a), although certain tumor types presented higher EORs (Figure 3b, Table 1). When the cases were stratified on the basis of fluorescence intensity, those without fluorescence had a lower rate of GTR (Figure 3c).

### 3.3. Side Effects

As described in previous studies, complications are rare in pediatric patients with 5-ALA-guided surgery [8,15]. Most complications, such as posterior fossa syndrome and neurological deterioration, were related to the resection per se, in which the strategy can be more aggressive and lead to greater EOR by the positive fluorescence [2,24,36]. The postoperative elevation in ALT and AST enzymes, compared with the preoperative levels, was discussed. Although there was a mild increase, it remained below the threshold for abnormality, with median values remaining within the normal reference range [34].

In the last 5 years, only one four-year-old male with fourth ventricle anaplastic ependymoma was reported with severe complications [40]. On postoperative day two, the course was complicated by hepatopathy, thrombocytopenia, and spontaneous right frontal intracerebral bleeding with intraventricular extension and subdural hematoma, requiring emergency craniotomy and evacuation of the bleeding. Finsterer pointed out that the intracerebral bleeding should not be attributed to 5-ALA before ruling out hepatic encephalopathy, medication during the preoperative period, and vaccine-induced immune thrombotic thrombocytopenia [42]. Thus, the safety of 5-ALA in pediatric patients has been preliminary established in more than 200 cases.

## 4. Discussion

Over the past five years, approximately one hundred additional cases of pediatric brain tumor resection via 5-ALA have been reported. These cases consistently followed the established protocols—5-ALA at a dose of 20 mg/kg administered by oral 3–6 h prior to surgery, with dexamethasone optionally administered several days preoperatively [2,7,8,9,10,11,12,13,14,15,23,24,25,27,28,30,31,32,33,36,37,38,39,40,41,42,43,44,45,46]. The disease spectrum primarily included medulloblastoma (MB), pilocytic astrocytoma (PCA), ependymoma, glioblastoma multiforme (GBM), anaplastic ependymoma (AE), and other tumors. The homogeneity in both the intervention and disease types has facilitated the preliminary analysis of data for certain tumor categories.

Moreover, some tumors presented results that were inconsistent with previous retrospective analyses, which diminishes the credibility of certain prior conclusions. Therefore, we re-evaluate the scope and effectiveness of 5-ALA use in pediatric brain tumors in this review.

### 4.1. Variation in the Fluorescence Rate and Utility Across Tumor Types

#### 4.1.1. Medulloblastoma and Pilocytic Astrocytoma

Medulloblastoma (MB) and pilocytic astrocytoma (PCA) are the most common CNS tumor types in pediatric patients [1,10]. Due to the high malignancy of MB and its propensity to infiltrate surrounding tissues, the use of 5-ALA as an adjunct in surgical enhancement is both justified and critical. The usefulness of 5-aminolevulinic acid (5-ALA) in treating medulloblastoma was first demonstrated by Eicker [10]. A total of 41 cases of MB have been reported, 16 of which (39.0%, including 9 weak) showed positive fluorescence. Among the 25 cases assessed for utility, 10 were deemed useful. Skjøth-Rasmussen et al. utilized 5-ALA-guided resection of an MB in the posterior fossa [27]. Although complete removal of the residual tumor was not achieved in the secondary surgery, the fluorescence facilitated maximal resection along the brainstem borders, thus allowing a less aggressive approach in postoperative radiation therapy [27].

Notably, over the past decade, a higher incidence of “fluorescence-positive” cases in medulloblastoma (MB) has been reported only in studies by Skjøth-Rasmussen (1/1) and Labuschagne (8/9, including 4 weak cases). In contrast, other studies involving multiple MB cases, such as Roth et al. (0/2), Goryaynov et al. (1 weak/6), Schwake et al. (0/3), and Milos et al. (0/4), have reported minimal or no fluorescence-positive findings [2,11,31,34]. Labuschagne used the estimated cutting time as the anchor point for 5-ALA administration rather than the start of surgery [36]. This variation in administration timing may partially explain these differences in results. Additionally, Labuschagne et al. reported a strong correlation between Ki-67 levels and fluorescence intensity in MB, with patients with Ki-67 levels above 70% exhibiting strong fluorescence and those below 50% exhibiting vague or no fluorescence. In vitro high accumulation of protoporphyrin IX (PPIX) induced by 5-ALA in medulloblastoma has been described by Briel-Pump et al., which is associated with low ferrochelatase expression and activity [47]. While with the high expression of the ABCG2 transporter protein (CD338), the accumulation of PPIX in MB appears to be lower than that in glioblastoma (GBM) [47]. The molecular mechanisms influencing PpIX suggest that the fluorescence mechanism in MB may differ across various subtypes, resulting in a mixed fluorescence rate in MB. Furthermore, selection bias in retrospective studies should also be considered.

As the most frequently reported type of tumor, PCA has a low fluorescence rate (11/42, 26.2%) and limited usefulness (7/14, 50%). Both PCA and MB typically differ from the surrounding brain tissue, allowing GTR to be achieved under the standard white light, thus minimizing the utility of using 5-ALA in PCA and MB [9,24,31,41,46]. As Nizolin et al. reported, the use of metabolic navigation with 5-ALA in PCA did not affect the resection volume since individual parts of the tumor visualized with 5-ALA were identified during microscopy in the visible light spectrum [41]. However, the utility of 5-ALA in the recurrent resection should not be dismissed, considering its potential for enhancing complete tumor removal [27].

The limited utility of 5-ALA was similarly reflected in pilomyxoid astrocytoma (PMA). For PMA, the fluorescence rate was 54.5% (6/11, including 2 weakly positive cases), and all the cases reported unhelpful fluorescence (2/2). Milos et al. observed “vague” fluorescence in one PMA case under the surgical microscope intraoperatively, which was not sufficiently useful to guide resection [2]. Schwake et al. did not observe fluorescence in PMA and noted that the in vitro detection of the PpIX concentration in this case was lower than that in other tumors with weak fluorescence [34].

The first pediatric brain tumor case in which 5-ALA was used to guide resection was reported by Ruge et al. in 2009 in a 9-year-old female with pleomorphic xanthoastrocytoma (PXA) [23]. To date, 50% of PXA cases have shown positive fluorescence (3/6, with 1 weakly positive), and 40% of the cases were considered useful (2/5), which is consistent with the findings of previous studies.

#### 4.1.2. Glioblastoma and Anaplastic Astrocytoma

Following the approval of glioma treatment in adults, glioblastoma is regarded as one of the most promising tumors for extending the use of 5-ALA to pediatric applications. To date, 11 studies have been conducted in pediatric patients, reporting a total of 33 GBM cases. The tumor location was specified in 27 of these cases, with 24 being supratentorial and 3 infratentorial. Fluorescence was positive in 28 out of 33 cases (84.4%, 3 weakly positive). Surgical outcomes included GTR in 7 cases, NTR in 1, and STR in 5 out of 13 cases. Fluorescence was considered useful in 21 out of 24 cases (87.5%). The overall effectiveness is consistent with previous findings on GBM [2,7,11,15,24,25,26,29,30,32,37]. With respect to recurrent GBM, Milos et al. suggested that in recurrent tumors, both normal tissue, gliosis, reactive astrocytes, and tumor cells can exhibit nonspecific PpIX fluorescence, rendering fluorescence insufficient to guide tumor resection [2]. In contrast, Preuß previously argued that 5-ALA remains necessary in recurrent GBM, particularly given the improved survival outcomes observed following complete resection in pediatric patients [24]. Thus, the consensus is that 5-ALA is beneficial in primary GBM resection. In recurrent GBM, study on additional margin sampling is needed to verify the likelihood of surrounding normal tissue being resected. Thus, the consensus is that 5-ALA is beneficial in the resection of primary GBM. In recurrent GBM, studies on margin sampling of resected tissue can be supplemented to verify the likelihood of surrounding normal tissue being resected.

Fluorescence was reported in 13 cases of anaplastic astrocytoma, with 10 cases showing strong fluorescence and 2 cases exhibiting weak fluorescence (one case did not report fluorescence status). The usefulness rate was 57.1% (4/7). This can be explained by the malignancy of anaplastic astrocytoma and the correlation between malignancy and fluorescence intensity. Further studies on AA are warranted.

#### 4.1.3. Ependymoma

Ependymoma demonstrated a high fluorescence rate, with 21 out of 25 cases showing fluorescence (including 3 weak cases), and usefulness was reported in 14 out of 24 cases (58.3%). The majority of authors agree that the use of 5-ALA in ependymoma is beneficial. Labuschagne suggested that the routine use of 5-ALA is likely to be valuable in the resection of CNS tumors in children with suspected or confirmed ependymoma [36].

Among the 12 cases of anaplastic ependymoma, 11 exhibited strong fluorescence, indicating a high fluorescence rate. Thus, 5-ALA could be a promising adjunct for ependymomas and anaplastic ependymomas.

#### 4.1.4. PNET

We included tumors reported as PNET, neuroblastoma, and ganglioneuroblastoma. Among the 12 cases, 5 were fluorescence-positive (41.7%, including 2 weak cases). All 8 cases with reported tumor locations were supratentorial. Due to the limited data, an analysis of EOR was not feasible. Among the 9 cases that reported utility, 4 considered 5-ALA to be effective. In personal communication from Ruge et al., a female patient diagnosed with PNET achieved complete resection with no evident tumor at 21 months postoperatively but experienced recurrence at 30 months [15]. The prolonged survival period suggests the potential utility of 5-ALA. However, further research focusing on survival outcomes rather than just GTR rates is needed.

#### 4.1.5. Other Rare Tumors

Other tumors were reported in fewer than 10 cases, including oligodendroglioma (8 cases), ganglioglioma (7 cases), diffuse astrocytoma (6 cases), and DNET (5 cases), with the remaining tumors having fewer than 3 reports. Among the WHO Grade III-IV tumors, one atypical teratoid/rhabdoid tumor (AT/RT) and two CNS embryonal tumors presented positive fluorescence, whereas choroid plexus carcinoma (0/1) and diffuse intrinsic pontine glioma (DIPG) (0/1) presented negative fluorescence. Among the low-grade brain tumors with WHO Grade I-II, only one DNET in the left occipital lobe and oligodendrogliomas (2/8, 2 with weak fluorescence not included) presented strong fluorescence, whereas the others, including atypical meningioma, diffuse astrocytoma, DNET, ganglioglioma, glioneuronal tumor, oligodendroglioma, and plexus papilloma, presented vague or no fluorescence.

Labuschange et al. reported the first 5-ALA-guided resection in AT/RT, which showed strong fluorescence and high utility of 5-ALA [2,9,29,34,36,37]. The utility of 5-ALA in meningiomas is ambiguous. A male with meningeal sarcoma was reported and achieved GTR under the guidance of intense fluorescence, which enabled the infiltrated dura mater and tumor tissue infiltrating the normal brain parenchyma to be distinguished. Bernal et al. reviewed the usefulness of 5-ALA in adult meningiomas for detecting tumor fragments that may remain in the cerebral bed or the walls of the dural sinuses and go unnoticed [12]. However, Milos et al. reported one atypical meningioma (WHO grade II) with vague fluorescence, which is not sufficiently useful to guide resection [2].

Only 2 cases with astroblastoma were reported separately by Agawa and Fudaba, both of which showed strong fluorescence. Price et al. reported an adult case of astroblastoma recurrence 21 years after gross-total resection and radiation, with the gene alternation as in-frame *MN1*::*BEND2* fusion transcripts, which was also found in one pediatric case [48]. Although the sample size was small, two pediatric cases and one case of astroblastoma originating in childhood, reported in adults, all exhibited strong 5-ALA fluorescence and achieved GTR. These findings suggest that 5-ALA may be a valuable tool for improving outcomes for this rare tumor type.

### 4.2. Mechanism and Determinants of 5-ALA-Induced Fluorescence

5-ALA is an endogenous precursor of protoporphyrin IX and plays a central role in heme biosynthesis. In healthy cells, PpIX is converted into heme by ferrochelatase, the rate-limiting enzyme in this process. However, in tumor cells, which are deficient in ferrochelatase, the administration of exogenous 5-ALA leads to the accumulation of PpIX. This accumulation has been experimentally observed both in vitro and in vivo within malignant tissues [17]. The blood-brain barrier, which is typically impermeable to 5-ALA, is disrupted in high-grade gliomas (HGGs), increasing the biodistribution of 5-ALA within tumor cells. The negative feedback control by heme on upstream enzymes is also abolished, further increasing PpIX biosynthesis. When irradiated with a blue light source (wavelength 375–410 nm), PpIX emits red fluorescence, which can be exploited during fluorescence-guided surgery to differentiate tumor tissue from normal brain tissue [49].

To explain the heterogeneity of fluorescence rates across different tumors, several factors have been proposed, including tumor malignancy (WHO grade), the concentration of PpIX in tumor tissue, patient age, and tumor location. Moreover, Traylor et al. explored the potential mechanisms of 5-ALA-induced glioma tissue fluorescence by reviewing related molecular and metabolic mechanisms [49]. Overall, the enhanced fluorescence is associated with low expression of ferrochelatase, overexpression of ABCB6, low expression of ABCG2, the accumulation of heme synthesis-related metabolites caused by IDH1 mutations, and the tumor microenvironment. Clinical manifestations may influence the occurrence of fluorescence through these mechanisms.

The malignancy of the tumor was considered the most important factor that affects the fluorescent rate. The fluorescence signal was more commonly observed in malignant glial tumors. Kim et al. demonstrated this by evaluating the correlation between the fluorescence rate and WHO grade and revealed that the intensity of fluorescence during surgery significantly depended on the malignancy grade of the tumor (N = 15, *p* = 0.05), i.e., the higher the WHO grade was, the greater the likelihood of 5-ALA fluorescence [30].

PpIX plays a key role in 5-ALA-induced fluorescence. Schwake et al. demonstrated a strong association between intraoperative observations and spectrometric measurements of PPIX fluorescence in tumor tissue by measuring the fluorescence intensity of tumor samples ex vivo via spectroscopy and comparing it to the visible fluorescence grade. A visible fluorescence signal was observed in all tumors that had a clear threshold of PpIX concentration at 4 μg/mL [34].

Beez et al. reported that systemic metabolic factors in infants might have a more significant effect on the utility of ALA than tumor-specific characteristics. Additionally, glioblastomas occurring in infancy are known to differentially express certain genes compared with pediatric and adult glioblastomas, which could further amplify the effects driven by systemic metabolic factors [25]. The difference in fluorescence rates was also reported by Wataya et al., who reported 11 cases with 8 different tumor pathologies and noted that positive fluorescence was not observed in patients younger than 4 years old [32].

The reliability of 5-ALA-induced fluorescence can be variable. Fluorescence enhancement of the ventricle wall has been observed in adults and may act as a confounder when operating in the brainstem or fourth ventricle. Labuschagne et al. also described this phenomenon in pediatric cases. In recurrent tumors, the surrounding edematous tissue has been hypothesized to contribute to false-positive fluorescence.

Preoperative factors can also influence the effectiveness of 5-ALA fluorescence. The preoperative use of antiepileptic drugs (AEDs) is common but has not been widely reported in most cases. Goryaynov et al. reported that visible fluorescence was significantly more common in patients not using AEDs than in those with preoperative AED intake (*p* = 0.046) [37,44]. One case report indicated that vomiting impaired the fluorescence efficacy [26], whereas Milos et al. described the nasogastric administration of 5-ALA under sedation for the first time, suggesting that it could be considered as a standard procedure [2]. Variations in intraoperative imaging devices have not received much attention, but Kamp et al. reported discrepancies in imaging results among different fluorescence devices, leading to the call for the development of equipment for the intraoperative quantification of PpIX fluorescence intensity [18].

Gadolinium enhancement and 5-ALA enhancement were commonly regarded as tumor imaging markers, but the correlation between them has not been thoroughly analyzed or emphasized. Labuschagne et al. reported a case of classic medulloblastoma with minimal gadolinium enhancement but strong 5-ALA fluorescence, concluding that contrast enhancement alone is not a reliable predictor of intraoperative fluorescence, whether positive or absent [36]. This discrepancy can be attributed to the differing metabolic pathways of gadolinium and 5-ALA. While both gadolinium and 5-ALA play crucial roles in brain tumor diagnosis, the consistency between them requires further investigation.

### 4.3. Limitations

The limited amount of statistical data made it unsuitable to conduct a meta-analysis to combine effect sizes. In addition, the inclusion of gray literature was insufficient in this review.

Some studies have shown negative results, whereas others, such as Labuschagne et al., reported positive outcomes. Importantly, Labuschagne administered the 5-ALA approximately 4 h prior to the cutting time, which differs from the standard practice. This discrepancy may indicate heterogeneity in this study design and other variables. Additionally, as a retrospective study, the tumor classification did not align with the latest WHO 2020 standard, leading to missing molecular and immunological characteristics, which rendered further analysis unavailable.

Given the demonstrated efficacy, a randomized controlled trial is recommended for pediatric HGG. Additionally, in future studies, a unified usefulness evaluation checklist and fluorescence grading system should be agreed upon. The molecular mechanisms mentioned by Traylor et al. could also be further validated in clinical samples or assessed through gene expression analysis to determine whether they align with the fluorescence patterns observed in various pediatric gliomas.

As with the retrospective survey conducted by Stummer, a follow-up study incorporating postoperative survival outcomes after fluorescence-guided surgery (FGS) would be valuable [26]. Additionally, sample bias should be carefully considered, and negative data should also be reported.

### 4.4. Future Research

Given the incomplete reporting of molecular subtypes across various tumor types, a retrospective evaluation of 5-ALA efficacy in different subtypes is currently not feasible. In low-grade gliomas, increasing the oral dose of 5-ALA to increase tissue PpIX concentrations may be considered, but careful attention must be given to patient age (at least 3 years old) and the integrity of the blood-brain barrier [50]. Although gadolinium enhancement on imaging is considered unrelated to 5-ALA fluorescence, other imaging modalities in adults, such as MET-PET, have demonstrated correlations with 5-ALA and Ki-67, suggesting the need for further exploration in pediatric populations. Black et al. developed machine learning algorithms capable of predicting tumor margins based on 5-ALA fluorescence in adults [51]. Extending similar research to pediatric 5-ALA postoperative samples could lay the foundation for real-time intraoperative identification of tumor margins using machine learning.

## 5. Conclusions

In general, 5-ALA fluorescence rates are positively correlated with tumor malignancy. No significant adverse effects directly attributed to 5-ALA have been reported, providing preliminary evidence of its safety in pediatric patients. Prospective clinical trials as well as smaller comparative studies focusing on dose and administration timing adjustments are warranted. However, long-term survival data remain rare, and the effectiveness of 5-ALA in improving survival curves and progression-free survival has not been established. However, before discontinuing the use of 5-ALA due to uncertainties regarding long-term benefits and inconsistent fluorescence rates, it is important to consider its role in enhancing resection rates and its potential for improving PFS. Therefore, retrospective analyses of past data across centers remain highly valuable and should include patient survival outcomes, tumor molecular subtypes, and a reassessment of the utility of 5-ALA.

## Figures and Tables

**Table 1:** Demographic characteristics of the included studies.

Author	Year	Country/Region	Design	No. of Patients	No. of Newly Reported Patients *	Age (Years)	Gender	DOI
Ruge et al. [23]	2009	USA	case report	1	1	9	F	10.3171/2009.6.PEDS08428
Eicker et al. [10]	2011	Germany	case report	1	1	15	F	10.1055/s-0030-1252010
Preuß et al. [24]	2013	Germany, France	case series	18	18	range 3–18; average age = 10.94 ± 4.67	13M; 5F	10.1007/s00381-013-2159-8
Barbagallo et al. [7]	2014	Italy	case series	3	3	range 8–18; average age = 12.67 ± 5.03	1F; 2M	10.3109/02688697.2014.913779
Beez et al. [25]	2014	Germany	case series	16	16	range 1–16; average age = 9	10F; 6M	10.1007/s00701-014-1997-9
Stummer et al. [26]	2014	Germany, Spain, Denmark, Ireland, Italy, Switzerland	case series	78	60	range 1–17; median age = 13	NA	10.1007/s00701-014-2234-2
Bernal García et al. [12]	2015	Spain	case report	1	1	7	M	10.1007/s00381-015-2703-9
Skjøth-Rasmussen et al. [27]	2015	Denmark	case report	1	1	9	M	10.1007/s00381-015-2762-y
Sysoev et al. [28]	2016	Russia	case series **	20	20	range 3–18	NA	10.1007/s00381-016-3044-z
Burford et al. *** [14,29]	2017	UK	case series **	6	6	NA	NA	10.1093/neuonc/now293.039
	2018	UK	case series **	10	4	range 1.6–15; median age = 6.5	NA	10.1093/neuonc/noy059.545
Kim et al. [30]	2017	Russia	case series	13	14 ^#^	range 3–17; average 9.23 ± 4.79	8F; 5M	10.17116/neiro201780751-57
Roth et al. [31]	2017	Israel	case series	14	14	range 4–19; average 11 ± 5	NA	10.1007/s00381-017-3371-8
Wataya et al. [32]	2017	Japan	case series **	11 ^##^	11	range 1–18	NA	10.1093/neuonc/nox168.988
Agawa et al. [33]	2018	Japan	case report	1	0	13	1F	10.1007/s00381-017-3714-5
Goryaynov et al. [11]	2019	Russia	case series	42	8	NA	NA	10.3389/fonc.2019.00830
Schwake et al. [34]	2019	Germany	case series	11	11	range 1–16; median = 10	6F; 5M	10.1007/s00701-019-04039-4
Zhang et al.(J Ruge’s personal communication) [15]	2019	USA	case report	3	3	range 8–15; median = 13	3F	10.1007/s11060-018-03004-y
Fudaba et al. [35]	2020	Japan	case report	1	1	6	F	10.2176/nmccrj.cr.2020-0028
Labuschagne et al. [36]	2020	South Africa	case series	19	19	range 2–12; average = 5	8F; 11M	10.1159/000511289
Labuschagne et al. [9]	2020	South Africa	case series	8	8	range 1–13; average = 6.1	2F; 6M	10.25259/SNI_246_2020
Labuschagne et al. [37]	2020	South Africa	case series	11	11	range 2–12; median = 4	7F; 4M	10.1016/j.wneu.2020.06.019
Beauchamp et al. [38]	2021	USA	case report	1	1	10	1M	10.3171/CASE20153
Maeda et al. [39]	2023	Japan	case report	1	1	14	1F	10.1007/s00381-022-05612-6
Milos et al. [2]	2023	Sweden	case series	14	14	range 4–17; median = 9	6F; 8M	10.1007/s00701-022-05360-1
Mui et al. [40]	2023	Ireland	case report	1	1	4	1M	10.1007/s00381-023-05846-y
Nizolin et al. [41]	2024	Russia	case report	1	1	9	NA	10.17650/1683-3295-2024-26-2-61-69

M male, F female, NA Not avaliable. *: For patients who were repeatedly included in multiple studies, the data were consolidated through manual cross-referencing of citations, timeframes, and clinical characteristics. **: reported as conference abstract. ***: Cases in two conference abstracts from Burford et al. have overlapped in time and clinical features. The revelent cases were merged into 10 patients. #: One patient underwent two surgeries and was subsequently diagnosed with AA and GB, thus being considered as two separate cases. ##: The pathological type of one case was not reported from Wataya et al.

**Table 2:** Summary of the fluorescence grade, EOR, utility of fluorescence, and location in different tumors (with n > 10).

Histology	No. of Cases	Fluorescence Grade				Extent of Resection				Utility of Fluorescence		Location		
		Strong	Weak	No	Total Fluorescence Reported	GTR	NTR	STR	Total EOR Reported	Useful	Total Utility Reported	Supratentorial	Infratentorial	Total Location Reported
PCA	42	6	5	31	42	10	2	4	16	3	9	7	14	21
MB	41	7	9	25	41	17	3	3	23	10	24	0	29	29
GBM	33	25	3	5	33	7	1	5	13	21	24	24	3	27
Ep	25	18	3	4	25	8	3	0	11	14	21	6	14	20
AE	13	10	2	1	13	4	1	3	8	4	4	6	2	8
AA	13	10	2	0	12	3	1	1	5	4	7	6	3	9
PMA	11	4	2	5	11	5	0	1	6	0	2	1	4	5
PNET	12	3	2	6	11	2	0	0	2	4	9	8	0	8

GTR gross total resection, NTR near total resection, STR subtotal resection, PCA pilocytic astrocytoma, MB medulloblastoma, GBM glioblastoma (formerly known as glioblastoma multiforme), Ep ependymoma, AE anaplastic ependymoma, AA anaplastic astrocytoma, PMA Pilomyxoid astrocytoma, PNET primitive neuroectodermal tumor.

## Data Availability

The raw data supporting the conclusions of this article will be made available by the authors on request.

## Supplementary Materials

The following supporting information can be downloaded at: https://www.mdpi.com/article/10.3390/cancers16213677/s1. Table S1: Search expression in the records identification. Table S2: Risk of bias and quality assessment of the studies included.
