# Supplementary material for: Utility and Safety of 5-ALA Guided Surgery in Pediatric Brain Tumors: A Systematic Review
Source: Cancers (Basel). 2024 Oct 30;16(21):3677. doi: 10.3390/cancers16213677 (PMC11545419; doi:10.3390/cancers16213677)
Supplement: Supplementary file 1 [file cancers-16-03677-s001.zip › cancers-3247670-supplementary.pdf]

Table S1. Search expression in the records identification.

Database	Search	Result	Date
Embase	('aminolevulinic acid'/exp OR 'aminolevulinic acid' OR '5-ala*' OR 'ala':ti OR '5-aminolevulin*' OR 'aminolaevuli*' OR 'aminolevuli*' OR 'δ-aminolevu*') AND ('rhabdoid tumor' OR 'atrt' OR 'at/rt' OR 'brain cancer' OR 'brain tumor' OR 'brain metastasis' OR 'glioma*' OR 'glioblast*' OR 'medulloblastoma*' OR 'mening*' OR 'ependymoma*' OR 'pilocytic*' OR 'intramedullar*' OR 'neuroblasto*' OR 'astroblastoma*' OR 'pnet*' OR 'dnet' OR 'neuroepithe*' OR 'neuroecto*')	2051	2024-08-01
Scopus	("aminolaevulinic" OR "aminolevulinic" OR TITLE-ABS (ala)) AND ("rhabdoid tumor" OR "atrt" OR "at/rt" OR "brain cancer" OR "brain tumor" OR "brain metastasis" OR "glioma*" OR "glioblast*" OR "medulloblastoma*" OR "mening*" OR "ependymoma*" OR "pilocytic*" OR "intramedullar*" OR "neuroblasto*" OR "astroblastoma*" OR "PNET" OR "DNET" OR "neuroepithe*" OR "neuroecto*") AND ("surgery" OR "neurosurgery" OR "resection") AND NOT ("breast" OR "bladder" OR TITLE-ABS-KEY ("photodynamic") OR TITLE-ABS-KEY (scar) OR TITLE (mouse) OR TITLE (murine) OR TITLE (rat) OR TITLE (cutan*)) AND (LIMIT-TO (SUBJAREA , "MEDI") OR LIMIT-TO (SUBJAREA , "NEUR") OR LIMIT-TO (SUBJAREA , "PHAR"))	3043	2024-08-01
PubMed	("aminolaevulinic" OR "aminolevulinic" OR "ALA") AND ("rhabdoid tumor" OR "atrt" OR "at/rt" OR "brain cancer" OR "brain tumor" OR "brain metastasis" OR "glioma*" OR "glioblast*" OR "medulloblastoma*" OR "mening*" OR "ependymoma*" OR "pilocytic*" OR "intramedullar*" OR "neuroblasto*" OR "astroblastoma*" OR "PNET" OR "DNET" OR "neuroepithe*" OR "neuroecto*")	1580	2024-08-01
Proquest	(aminolaevulinic OR aminolevulinic) AND ("rhabdoid tumor" OR "atrt" OR "at/rt" OR "brain cancer" OR "brain tumor" OR "brain metastasis" OR "glioma*" OR "glioblast*" OR "medulloblastoma*" OR "mening*" OR "ependymoma*" OR "pilocytic*" OR "intramedullar*" OR "neuroblasto*" OR "astroblastoma*" OR "PNET" OR "DNET" OR "neuroepithe*" OR "neuroecto*") NOT ("mouse" OR "mice" OR "cutan*" OR "murine" OR "rat" OR "photodynamic" OR "ultrasound") + fliter selected manually	356	2024-08-01

Table S2. Risk of bias and quality assessment of the studies included.

Author	Year	Country/Region	Q1	Q2	Q3	Q4	Q5	Q6	Q7	Q8	Q9	Q10
JBI Critical Appraisal Checklist for Case Series												
Preuß et al.	2013	Germany, France	N	Y	Y	N	N	Y	Y	Y	N	Y
Barbagallo et al.	2014	Italy	N	Y	Y	N	Y	Y	Y	Y	N	Y
Beez et al.	2014	Germany	Y	Y	Y	Y	N	Y	Y	Y	N	Y
Stummer et al.	2014	Germany, Spain, Denmark, Ireland, Italy, Switzerland	Y	Y	N	Y	unclear	Y	unclear	Y	N	Y
Sysoev et al.	2016	Russia	N	Y	Y	Y	N	unclear	Y	Y	N	Y
Burford et al.	2017	UK	N	Y	Y	N	N	N	Y	Y	N	Y
Burford et al.	2018	UK	N	Y	Y	N	N	N	Y	Y	N	Y
Kim et al.	2017	Russia	Y	Y	Y	Y	Y	Y	Y	Y	N	Y
Roth et al.	2017	Israel	Y	Y	Y	Y	Y	Y	Y	Y	N	Y
Wataya et al.	2017	Japan	N	Y	Y	N	N	N	Y	Y	N	Y
Goryaynov et al.	2019	Russia	N	Y	Y	Y	Y	Y	Y	Y	N	Y
Schwake et al.	2019	Germany	N	Y	Y	Y	Y	Y	Y	Y	N	Y
Labuschagne et al. (Posterior Fossa Tumours)	2020	South Africa	Y	Y	Y	Y	Y	Y	Y	Y	N	Y
Labuschagne et al. (brainstem gliomas)	2020	South Africa	Y	Y	Y	N	N	Y	Y	Y	N	Y
Labuschagne et al.(Supratentorial)	2020	South Africa	Y	Y	Y	N	N	Y	Y	Y	N	Y
Milos et al.	2023	Sweden	Y	Y	Y	Y	Y	Y	Y	Y	N	Y
JBI Critical Appraisal Checklist for Case Reports												
Ruge et al.	2009	USA	Y	Y	Y	Y	Y	Y	Y	Y	/	/
Eicker et al.	2011	Germany	Y	Y	Y	Y	Y	Y	Y	Y	/	/
Bernal García et al.	2015	Spain	Y	Y	Y	Y	Y	Y	Y	Y	/	/
Skjøth-Rasmussen et al.	2015	Denmark	Y	Y	Y	Y	Y	Y	Y	Y	/	/
Agawa et al.	2018	Japan	Y	Y	Y	Y	Y	Y	Y	Y	/	/
Zhang et al.	2019	USA	Y	N	N	Y	Y	Y	N	Y	/	/
Fudaba et al.	2020	Japan	Y	N	Y	Y	Y	Y	unclear	Y	/	/
Beauchamp et al.	2021	USA	Y	unclear	Y	Y	Y	Y	N	Y	/	/
Maeda et al.	2023	Japan	Y	Y	Y	Y	Y	Y	Y	Y	/	/
Mui et al.	2023	Ireland	Y	Y	Y	Y	Y	Y	Y	Y	/	/
Nizolin et al.	2024	Russia	Y	unclear	Y	Y	Y	Y	Y	Y	/	/

Y yes, N no. All studies were included.

Numbers Q1-Q10 in case series: Q1, were there clear criteria for inclusion in the case series? Q2, was the condition measured in a standard, reliable way for all participants included in the case series? Q3, were valid methods used for identification of the condition for all participants included in the case series? Q4, did the case series have consecutive inclusion of participants? Q5, did the case series have complete inclusion of participants? Q6, was there clear reporting of the demographics of the participants in the study? Q7, was there clear reporting of clinical information of the participants? Q8, were the outcomes or follow up results of cases clearly reported? Q9, was there clear reporting of the presenting site(s)/clinic(s) demographic information? Q10, was statistical analysis appropriate?

Numbers Q1-8 in case reports: Q1, were patient's demographic characteristics clearly described? Q2, was the patient's history clearly described and presented as a timeline? Q3, was the current clinical condition of the patient on presentation clearly described? Q4, were diagnostic tests or assessment methods and the results clearly described? Q5, was the intervention(s) or treatment procedure(s) clearly described? Q6, was the post-intervention clinical condition clearly described? Q7, were adverse events (harms) or unanticipated events identified and described? Q8, does the case report provide takeaway lessons?
